# The Hospitals and Dispensaries in Burma

**Published:** 1901-04-27

**Authors:** 


### THE HOSPITALS AND DISPENSARIES IN BURMA.

Five new hospitals were opened in Burma during the year 1899, so that the total rose from 105 to 110. The hospital at Lemyethona was constructed in consequence of the local authorities representing the urgent need for medical aid to alleviate the sufferings stated to be caused by various complaints, especially fevers, rife in that part of the Henzada district. A building, which had been constructed for a school, had to be given up to accommodate the hospital, furniture and patients, and the hospital was fit for use in May. The total number of police hospitals remained the same as in the previous year, the admissions rising from 15,555 to 16,273; but, unfortunately, the death rate also rose considerably. In 1898 it stood at 10·84, in 1899 it was 17·42. The Inspector-General of Civil Hospitals considers that these unsatisfactory figures are due to the detention of battalions for too long a period in unhealthy stations, as well as to defective housing. The aggregate number of patients treated throughout the year in all the dispensaries and hospitals throughout Burma was 818,039, which was an increase of 19,000 over the total of the year before. The largest increase was in the number of children brought by their parents for medical or surgical advice. There were also more male patients than before, but the number of women decreased by 20. This decline in female patients, which was especially marked amongst the Burmans, of whom there were 3,000 less than in 1898, is attributed to the lack of separate accommodation provided for them, and to the want of consideration shown to them by examining them in more or less public places. The freedom allowed to women in Burma appears to have led to the presumption that separate waiting accommodation is unnecessary, and that women attending for treatment or bringing children for that purpose can be expected to wait their turn with the men, and that examination and questioning of females can be carried on without objection being taken to it. At Rangoon some progress in this matter has been made because, three years ago, the male and female out-patients were not separated at all; but even now the two sexes are in full view of each other from their respective waiting enclosures, and the accommodation for female out-patients is most unsatisfactory. The opinion of those who have the welfare of the hospitals at heart is that a perfectly separate waiting-room—not an enclosure—a well appointed room for examining cases, and a well-trained out-patient nurse would soon render the gynæcological department more popular. As the out-patient department is the main recruiting ground for in-patients, the numbers of in-patients and of important gynæcological operations suffer accordingly. Speaking of operations in general, though there has been an increase in the year under consideration, with a slightly larger ratio of successes, the Inspector-General reports that he is by no means satisfied with the progress made in this direction. Those civil surgeons who are in charge of jails in particular, are too apt to leave the hospital work in the hands of subordinate medical officers.

With regard to diseases in need of operation, it is said that cases of stone and cataract are rare; but the Inspector-General of Civil Hospitals thinks that they are not so rare as reported, and could easily be found if sought for, and that cataract is frequently met with in the villages of the Meiktila district. These are more easily discovered by the officer on vaccination duty, and, where practicable, the sufferers should be induced to come to the hospital to see what can be done for them. A successful cataract operation, resulting in the restoration of eyesight, appeals more to the imagination of the people than any other operation, and where such operations are successfully performed the popularity and usefulness of a hospital or dispensary will be greatly enhanced. As to the supply of medicines, surgical instruments, beds, bedding, &c., the hospitals are well equipped, but the ordinary surgical work of the Burman medical institutions leaves much to be desired. Wounds and sores are frequently dressed in an open verandah, where there is no privacy, or upon the earthen floor of an enclosed corner with no attempt at antiseptic precautions beyond the application of some carbolic oil. The usual douches, which are cheap, and can usually be obtained locally, are wanting, and a nailbrush is an exception, even in the operating rooms of the hospitals.

The most serious epidemic which visited Burma during 1899 was one of small-pox. Its ravages were especially felt in Rangoon and the surrounding delta districts, and the cases numbered 1800 as compared with 651 in 1898. The disease was of a very virulent type, and hæmorrhagic cases occurred with considerable frequency, and were nearly always fatal. The first case was admitted in the middle of January, and for a couple of months small-pox was very prevalent. In April the epidemic abated rapidly, and by the end of May it had practically ceased to exist. Amongst the vaccinated cases, with a few exceptions, the disease ran a mild course, and the death-rate was comparatively low. Considerable difficulty was experienced in obtaining enough nurses and attendants to wait on the large number of patients, and in spite of paying higher wages it was not uncommon to find many of the ward boys and mehters absconding during the night from the native hospital. It is hoped that the passing of the Burma Vaccination Law Amendment Bill will tend to check ravages of this virulent disease. Leprosy, which had shown a marked tendency to decrease, rose again in 1899, and beri-beri was responsible for a number of cases. Its prevalence in Rangoon has from time to time attracted some attention, and inquiries have been made so as to learn, if possible, the origin of the ailment. The poison which produces beri-beri is believed to reside in the soil, and to be fostered by damp and high temperature. The disease is admittedly most prevalent in Rangoon during the months of October, November, December, and January, and the Hindus, especially of the coolie class, are attacked out of all proportion to other castes. Of these Hindus, by far the greatest number of patients are men. As far as investigation has gone at present, it appears that the curve of prevalence of beri-beri corresponds very closely with the immigration curve of coolies from Southern India. These coolies, upon arrival, generally go into the districts to reap the paddy; they live in houses of poor construction, mat walls and thatched roofs, and sleep on the damp ground instead of on a raised wooden platform, as the Burmans do; their diet, though sufficient in quantity, is deficient in nitrogenous elements, consisting chiefly of rice, vegetables, and dried fish. For a year they do not seem affected, then they suffer from fever, which they never really get over, but become weaker and weaker. When admitted to hospital, if the case is not too far gone, they rapidly improve on a nitrogenous diet, especially good results being obtained from the administration of bone marrow. Captain Barry, I.M.S., has made a special study of this disease, and he is of opinion that it is endemic in the delta districts of Burma, but that the Burmans are less liable to attack than the Hindus because their conditions of life are healthier.

There was considerable increase both in the total income and expenditure of dispensaries in Burma during 1899. The income is higher from nearly every source, especially the contributions from Government as "special allowance" and from "municipal funds." The amount collected by subscriptions has been expended rather more liberally than in the previous year, but it helped to secure many additional comforts for the patients. Those who needed special attention were provided with nurses and attendants, and where particular articles of diet, not allowed by the hospital, such as iced drinks, wines, &c., seemed necessary, the subscriptions defrayed the expense. Also hospital walls were brightened by pictures and ornaments, and easy chairs and bedside tables purchased where required. Free gifts of clothing were also given upon their discharge to poor patients whose clothing had been destroyed, because of infection, from the same fund.

